# Patient voice on management of facial dermatological adverse events with targeted therapies: a qualitative study

**DOI:** 10.1186/s41687-019-0116-3

**Published:** 2019-05-02

**Authors:** Kaori Yagasaki, Hayato Takahashi, Takeshi Ouchi, Jun Yamagami, Yasuo Hamamoto, Masayuki Amagai, Hiroko Komatsu

**Affiliations:** 1 Faculty of Nursing and Medical Care, Keio University, 35 Sinanomachi, Shinjuku-ku, Tokyo, 160-8582 Japan; 2 Department of Dermatology, Keio University School of Medicine, 35 Sinanomachi, Shinjuku-ku, Tokyo, 160-8582 Japan; 3 Keio Cancer Center, Keio University School of Medicine, 35 Sinanomachi, Shinjuku-ku, Tokyo, 160-8582 Japan

**Keywords:** Dermatological adverse events, Integrated care, Patient-centered care, Qualitative study, Quality of life, Targeted therapies

## Abstract

**Background:**

With the increased use of targeted therapies in oncology, dermatological adverse events (dAEs) have drawn attention. Because the face is crucial for human identity and social interactions, facial dAEs have significant impact on a patient’s quality of life. This study aimed to explore patients’ experience with regard to the management of targeted oncological therapy-induced facial dAEs.

**Methods:**

In this qualitative study, 20 patients at a university hospital in Japan with advanced/metastatic cancer and targeted therapy-induced facial dAEs were individually interviewed to collect data. Thematic analysis was used to analyze the data.

**Results:**

Patients with cancer and targeted oncological therapy-induced facial dAEs who were referred to the Department of Dermatology had certain expectations from specialist services. Three key themes were identified: professional input and advice, empathetic commitment to individual management, and integrated care across specialties.

**Conclusions:**

The referred patients with cancer and facial dAEs needed more in-depth information and advice from dermatological services and were reassured by the empathetic commitment to individual management in integrated care across specialties. These findings suggest that attention to the patient’s perspective with a “sick person first” attitude and a collaborative effort across different specialties is important to minimize the effects of facial dAEs on the quality of life of patients with cancer.

## Background

With the increased use of targeted therapies in oncology, dermatological adverse events (dAEs) have been commonly reported. In particular, treatment with epidermal growth factor receptor (EGFR) inhibitors may result in the manifestation of dAEs, such as papulopustular rash, xerosis, and pruritus, in more than 80% of patients; this can lead to dose reduction or therapy discontinuation [1]. EGFR inhibitor reportedly induced papulopustular eruption/acneiform rash, which developed a bacterial superinfection that made patients visually disturbing in 21% [2] and 29% [3] of the total patients treated.

Proper management is essential to minimize patient discomfort during cancer therapy [4, 5]. Although there are no standard therapies for targeted therapy-induced dAEs, treatments, such as skin moisturizers and topical ointments, have been recommended to prevent and reduce the symptoms [6]. Because self-administered oral chemotherapy is increasingly available, the responsibilities of side-effect monitoring and symptom management have recently shifted from the healthcare provider to the patient [7].

The literature suggests that the development of skin rash is associated with the effect of EGFR inhibitors [8]. However, the effects on the quality of life (QOL) should be also considered. A recent survey revealed that skin toxicities were rated as the most impactful adverse events in cancer treatment, along with nausea/vomiting, higher than fatigue and hair loss [9]. Furthermore, when the patient’s QOL related to dAEs was compared between targeted and non-targeted therapies, targeted therapies were found to be associated with more negative effects on the patient’s dermatology-specific QOL (the emotional and functional domains of QOL) [10].

Previous qualitative studies reported that treatment-induced skin toxicities disrupted patients’ daily lives and negatively affected their self-image, social engagement, and intimate relationships in addition to causing actual physical discomfort [11, 12]. A study on facial acne vulgaris suggests that a distorted self-image, particularly the facial image, causes psychosocial problems, including low self-esteem, anxiety, and depression, and negatively impacts social relationships [13]. Because the human face is more exposed in comparison to other parts of the body [14], it has a major significance in almost every aspect of human life [15]. In the present study, we focused on patients with cancer having targeted therapy-induced facial dAEs and aimed to explore their experience with regard to the management of dAEs.

## Methods

### Study design

This was a qualitative study of data collected from individual interviews. Thematic analysis, a method used to identify, analyze, and report themes within data [16], was used to explore the experiences of patients with advanced/metastatic cancer and targeted therapy-induced facial dAEs. The study design was approved by the Institutional Review Board of the Faculty of Nursing and Medical Care, Keio University (No. 266), and the Institutional Review Board of the Keio University School of Medicine (No. 20170252).

### Setting and participants

The inclusion criteria for selecting the participants were as follows: 1) patients with cancer having targeted therapy-induced facial dAEs (e.g., acneiform rash, xerosis, pruritus, and erythema), who were aged 20 years and older; 2) those who were able to monitor and care for their skin; and 3) patients without hearing or cognitive impairment, who were able to communicate and decide whether to participation or discontinue participation in the study. Patients with severe physical pain or discomfort were excluded.

Potential participants with advanced cancer were invited to participate in the study by their attending physicians during their visits to the Departments of Dermatology, Cancer Center, and Departments of Gastroenterology and Hepatology, Keio University Hospital in Tokyo. The physicians briefly explained the research outline and introduced the investigator (KY) to only patients who were interested in hearing the details from the investigator. Subsequently, the investigator and the patient met privately in a room that was different from the consultation room. Written and oral explanations of the objectives, methodologies (e.g., interview, recording), and voluntary participation were given to those who were interested in participating in the study. One patient withdrew before providing informed consent mainly because the interview was a burden owing to fatigue. All other participants (*n* = 20) provided oral and written informed consent to participate in the study and chose to be interviewed either on the day of recruitment or on the day of the next scheduled visit.

### Data collection

Informed consent from all 20 participants were obtained during consultation or in the interview room in the hospital on a date chosen by each participant. One of the investigators (KY) conducted the interviews using a semi-structured interview guide in the hospital between March and July 2018.

Based on a literature review of the multifaceted effects of EGFR inhibitor-related dAEs on QOL [5, 9, 10], living experience [11] of cancer patients receiving therapies, and discussion among the research team members, we developed a semi-structured interview guide. After the completion of the third, ninth, and 15th interviews, KY and another investigator (HK) reviewed the data and interview guide to ensure the quality of data and revised the interview guide to facilitate patients with advanced cancer to talk about their experience with facial dAEs. The Appendix presents the final version of the interview guide. We used purposive sampling based on the inclusion criteria in the present study. Open questions were used to obtain information on the participants’ feelings about cancer and facial dAEs in the introductory phase of the interview. The questions that followed were in line with the semi-structured interview guide. KY and HK discussed emerging themes, and data collection continued until the data reached saturation. The interviews were audiotaped and transcribed verbatim.

### Data analysis

The transcripts were reviewed and analyzed using thematic analysis. We used the qualitative research software, NVivo10®, to record and manage the data. KY reviewed the transcripts several times, anonymizing personally identifiable information. Adhering to the phases of thematic analysis [16], the following procedures were implemented: 1) KY independently and continuously, reviewed the transcripts; 2) data that were determined relevant were extracted from the entire data set and labelled with initial codes; 3) candidate themes were identified by repeatedly comparing and integrating individual codes and reporting patterns in the data; 4) themes were identified by reviewing candidate themes; 5) subthemes were determined; and 6) quotations were selected for use in the results.

To ensure the trustworthiness of the study, we adopted the following procedures: KY analyzed the data, and noted personal perceptions and subjectivity in parentheses. Then, HK reviewed the data with the initial codes and tentative subthemes. All the themes and subthemes were discussed, and final themes were established by KY and HK. After the completion of all interviews, the research team had two peer debriefings and email exchanges about the themes of study and the interpretations, including the opinions of the dermatologists (HT, TO, JY, and MA) and oncologist (HY). In consideration of the participants’ physical and psychological burdens, we did not perform a member check by asking participants to confirm the data, themes, and interpretations. After completing the identification of the themes and quotations that supported the themes, a professional translator translated them into English.

## Results

Twenty patients participated in the study: 6 (30%) were women; age range 38.0 – 87.0 (mean 63.7 years, median 65.5 years). Demographic and medical information is presented in Table 1. All patients had recurrent or metastatic disease. The interview duration was 18-55 min (mean 28.4 min, median 26.5 min). Patients with cancer having targeted therapy-induced facial dAEs who were referred to the Department of Dermatology had certain expectations from specialist services. Three key themes were identified.

**Table 1:** Patient Characteristics (*n* = 20)

Characteristics		
Age		
Mean (median), range	63.7 (65.5)	38.0-87.0
Gender	n	%
Female	6	30
Male	14	70
Employment status		
Full-time and part-time	10	50
Unemployed, retired	9	45
Housewife	1	5
Marital status		
Married	18	90
Single (including divorce/widowed)	2	10
Primary cancer diagnosis		
Lung	14	70
Colon	4	20
Laryngeal and pharyngeal	2	10
Cancer treatment		
Afatinib Maleate	6	30
Erlotinib	5	25
Panitumumab	4	20
Others (Gefitinib, Cetuximab, Osimertinib)	5	25

### Theme 1. Professional input and advice

#### Provision of in-depth information on disease outlook

Although participants were aware that skin toxicities might develop with EGFR inhibitors at the time of targeted-therapy initiation, uncertainty about the disease outlook was very stressful. Patients wanted a detailed explanation, including the duration of rashes and worst-case scenarios, from the dermatologist and the nurse as a specialist service.

One of the participants did not know that skin toxicities could have repeated cycles of exacerbation and improvement because the drug leaflet only described one cycle. She said, *“Looking at the graph, skin symptoms appear about every two weeks…I received it (the leaflet about targeted therapy and thought this (acne-like rash) would be healed (for two weeks). But the symptoms are getting even worse, and it appears here (in the cheek) too. It was a shock” (P1, female).* Clearly informing the patient of this possibility might have helped control her psychological status better.

Another participant did not know how to assess the severity of his symptoms and felt that self-identification was difficult even though he could gain information through the Internet or the leaflet. He said, *“I searched in the Internet, but I don’t know whether grade 1 or 2 is good or bad. It is difficult to understand it” (P14, male).*

If the disease outlook is known, participants can be prepared for treatment. One of the participants gradually gained her knowledge about the cycle of dAE through her experience: *“I feel much better if I know the outlook. When I suffered from dAE for the first time, I felt down because I was afraid that this condition would persist or even get worse. An uncertain future makes me uneasy. I was a little relieved this time because I roughly understood my own cycle” (P4, female).*

#### Proactive advice on a clue of improvement

Most participants recognized their individual roles for managing skin care at home by themselves. For example, one of the participants cared for his sick wife at home and was aware that he had to care for his own skin as well; he kept detailed records of his physical condition and changes in skin symptoms. He said, *“I need a treatment that is suitable for my lifestyle. Because it is not inpatient care provided by the nurse, I have to do it by myself at home” (P 11, male).*

Another participant was desperate for any help from a dermatological care specialist because of symptoms of widespread pain or itch on the face, nails, body, and extremities. He applied the prescribed ointment exactly as he was directed. When no improvement was observed despite thorough self-care, he felt hopeless: *“I don’t know what to do. I am really working hard at applying the ointments, but it doesn’t work at all” (P 5, male).*

In contrast, one of the participants said, *“Referring to the dermatologist was absolutely helpful” (P 4, female).* This woman was disappointed with her altered facial appearance that *“looked more than 10 years older”* and avoided going out. However, the most distress symptoms, redness around the eyes, and dryness-related wrinkles, were improved with the dermatologist’s treatment and the nurse’s advice on skin care.

### Theme 2. Empathetic commitment to individual management

#### Understanding the patient’s problem

High visibility of dAEs on the face had a great impact on the patients’ social lives. Both male and female patients often wore masks and glasses or avoided going outside because they were concerned about stares from other people suspecting contagious diseases or progression of cancer. One of the participants reported that his facial dAE was considered contagious by other individuals at a pool in a sports club: *“People whom I don’t know told me, ‘you have many lumps. Are you alright?’ They thought AEs were lumps. It was the most disappointing thing because I had to tell them every time that these were AEs of the anticancer drug” (P 17, male).*

Although participants did not actively intend to hide the fact that they had cancer, they did not appreciate that they were being assessed based on the affected face: *“(I was told that)‘you have something in the face’ that makes people (at workplace) uneasy. They think that the disease is progressing. They judged by this face instead of talking to me…” (P 7, male).* In contrast, one of the participants with dAE (grade 3) said that he did not care about his appearance much as long as he was recognized as a team member (at the workplace). Because people at his workplace did not treat him differently, he continued his work.

In these situations, some participants were reluctant to tell the physician because they were not sure whether it was the right topic to discuss during consultation: *“It will be helpful if the doctor listens to me (attentively). The patient has a little hesitation (to say something in front of a doctor)” (P 17, male).* They rarely have a chance to see outpatient nurses: “*Because of limited time of outpatient care, nurse involvement is limited” (P 8, female).*

Some participants lost a chance to share their concerns or problems with healthcare providers if they were not asked about them by the healthcare providers. Furthermore, one of the participants thought that truly understanding someone’s distress was difficult, even when he consulted with the physician: *“The doctor himself does not suffer. He can learn (the dAE) only through the book. Probably he cannot understand my suffering well. Maybe people cannot understand unless they go through it themselves” (P 7, male).*

#### Collaborative-seeking solution

One of the participants even wanted dose reduction of the anticancer drug owing to symptom severity. The dermatologist understood the patient’s concerns well and tried to find a solution together. He said, *“I think the dermatologist is doing his best. As he suggested multiple options, I tried six to eight different drugs. We are discussing to continue the use of one that was effective. It’s a trial-and-error approach” (P 13, male).*

Even though skin symptoms were not improved, participants were encouraged and supported to continue treatment if healthcare providers understood their distress and tried to find a solution together. Many participants believed that they should not rely on healthcare providers but collaborate with them by taking each role. One of the participants said, *“The nurse always told me keep hygiene and moisture very attentively. So, I am working hard on moisture and washing my face because that’s the only thing I can do by myself” (P 4, female).* Another participant said, *“I don’t think it works out if only the physician or the patient works. I believe both (should work together)” (P 13, male).*

Participants emphasized that even a subtle improvement and perceived empathy makes a major difference in their lives. One of the participants stated, *“If there is one improvement or even one symptom gets better, it is a pleasure. Really, even one symptom gets better, one terribly annoying thing was over” (P 4, female).*

### Theme 3. Integrated care across specialties

#### Healthcare providers’ “sick person first” attitude and collaboration

Most participants welcomed the referral offer from the oncologist: *“Honestly, I thought it would be better to be treated at the Department of Dermatology because they are specialists of dermatology and it is their specialty” (P 13, male).* Participants felt reassured that they would receive the best treatment and care, if healthcare providers around them shared patient information, including their condition and history of the disease, and worked together. One of the participants said, *“I am impressed that professionals (in different specialties) work as a multidirectional and attentive team” (P 6, male).*

One of the participants appreciated that different specialists discussed about his treatment. He felt secured by the integrated care across specialties, particularly with regard to good information exchange: *“I believe that information was given (from the pulmonologist to the dermatologist) and also to the radiologist. I was advised in many ways. (…) Of course, I feel secured because they discuss about my condition from various aspects even at the conference. I know it is for me” (P 17, male).*

One of the participants who received treatment at the hands of multiple physicians having different specializations felt that “the sick person first” attitude provided reassurance to continue treatment. She described, *“I feel something like ‘the sick person first’ in this hospital. Every time I come here, I feel relieved” (P 8, female).*

## Discussion

This is the first study to describe the perspectives of patients with cancer having targeted therapy-induced facial dAEs with regard to management strategies. It is known that EGFR inhibitor-induced dAEs have major negative effects on the QOL of patients with cancer [11, 12]. This study revealed that the high visibility of dAEs on the face caused significant distress owing to the unpredictability of the changes in dermatological symptoms, such as redness, pustule formation, bleeding, and dryness, in addition to having a significant impact on the patient’s social life (misinterpretation and prejudice from other people) and psychological status. Therefore, the patients needed more in-depth information and advice from dermatological services and were reassured by the empathetic commitment to individual management in integrated care across specialties.

The participants of the present study wanted more detailed information on disease outlook, including duration, severity, and possible improvement of dAEs. A previous study with patients undergoing oral chemotherapy reported that many patients feel helpless at home when they do not have enough information and knowledge about their treatment and symptoms [7]. In general, education on the self-management of dAEs is recommended both before and after initiation of cancer therapy [4, 5, 17, 18]. However, it is difficult for patients to predict the disease outlook because various types of dAEs may develop [1, 18], and exacerbation and improvement of symptoms often repeatedly occur after the initiation of EGFR inhibitors. A referral to dermatological departments is a step towards moving out of their current situation, where patients feel afflicted by dAEs amid a period of uncertainty due to cancer. Patients expect professional input at the Department of Dermatology, and with such information, they can be prepared for treatment.

The participants also wanted professional advice on finding indications for some improvement in their symptoms. They were disappointed if the medicines prescribed by the dermatologist or the skin care instructed by the nurse did not improve their symptoms. In contrast, the patients highly appreciated specialist services when the most distressful symptom was improved by the dermatologist’s treatment and the nurse’s advice on skin care.

To give the best advice to patients, healthcare professionals need to understand their problems. When healthcare providers are not attentive, some patients lose a chance to share their concerns or problems with healthcare providers. Identifying the patient’ true needs is the most challenging aspect of the treatment; the quality of the relationship can be therapeutic for the patient through the expression of openness, acceptance, and a caring attitude [19]. In such cases, patients no longer remain passive but move towards partnership [20]. The participants of this study recognized that they should take their role in the management of dAEs more seriously. Collaborative efforts with healthcare providers are important for patients. They emphasized that even a slight improvement and perceived empathy would be significant for them.

In this study, many patients appreciated integrated care, and one of them described it as a “sick person first” approach. The integrated care across specialties, particularly good information exchange, reassured the patients to continue treatment. The patients felt comfortable if their information, particularly with regard to the cancer treatment, was given to dermatological professionals when they were referred. In other words, some of the patients said that it would be stressful to explain the history of cancer treatment during dermatological consultation. In integrated care across specialties, collaboration between oncologists and dermatologists is important [21]. Nurses from the oncology department can promote integrated care by collaborating with not only oncologists but also dermatologists and outpatient nurses at the Department of Dermatology. Patient-centered care contributes to advancing harmonization between the healthcare provider and the patient on treatment plans, improving health outcomes, and increasing patient satisfaction [20, 22].

### Limitations

Because of the study design, there is the possibility that the selection of the participant population, the involvement of a single interviewer, and the sample being drawn from a single institution influenced the results of the study. We tried to focus on facial targeted therapy-induced dAEs in the interviews. However, patients with advanced cancer talked about symptoms other than dAEs and fear of cancer. The interview was conducted once for each patient. Some patients were tired because of a long waiting time at the outpatient clinic, and had short interview times. Thus, it was difficult to deepen the conversation about facial dAEs for some patients. In this study, the attending physician approached patients in the clinics to invite them to participate in the study. To minimize the effect of the attending physician’s involvement in recruitment, the investigator explained the details of the research and obtained informed consent from the patient in another room. However, a potential for coercion cannot be completely denied.

### Implications for clinical practice and future research

Patients expect healthcare providers to understand their problems and be proactively involved in individual management of facial dAEs. Good care encompasses patient-centered care, knowledge-based care, and skillful practice [23]. Nurses in the oncology department and outpatient nurses at the Department of Dermatology need to be sensitive to signs and symptoms of facial dAEs in individual patients and seek their solutions together, rather than having the patient go through this process alone. Advanced care for skin toxicities and empathetic communication skills are required to solve the patient’s problem. Future research should focus on effective methods of self-management at home to enhance the QOL of patients with cancer having targeted therapy-induced facial dAEs. Nurses in oncology departments can provide seamless patient information to dermatological professionals and promote patient-centered care. Patients can be reassured to continue treatment through these efforts.

## Conclusions

The present study revealed the perspectives of patients with cancer having target therapy-induced facial dAEs for the first time. The patients expected professional input and advice from dermatological services, including in-depth information on disease outlook and proactive advice on improvements in symptoms. Patients were reassured by the empathetic commitment to individual management in integrated care across specialties. Since dAEs might be an evidence of antitumor activity, patients are likely to prefer more efficacious cancer therapy and accept a higher probability of severe skin toxicities [9]. To minimize the effects of facial dAEs on the QOL in patients with cancer, it is important for healthcare providers to listen to the patients’ perspectives and understand individual problems and needs. With the “sick person first” attitude, healthcare providers can collaborate across different specialties to support patients with facial dAEs.

## Appendix

**Table 2:** Interview Guide

1. Does facial skin symptom impact on your daily living? Did your daily living, including interpersonal relationship, work, and hobbies, change because of facial dAEs? Would you talk about it?	
2. Do you feel that healthcare providers understand your needs and problems relevant to skin care at home?	
3. How do you feel about the referral to the dermatologist from the oncologist?	
4. What do you expect from healthcare providers regarding facial dAEs? Are you satisfied with the relationship with healthcare providers?	

Abbreviations: *dAEs* dermatological adverse events

## Abbreviations

dAEs: Dermatological adverse events
EGFR: Epidermal growth factor receptor
QOL: Quality of life
